# Dehydroepiandrosterone Sulfate in Diagnosing Mild Autonomous Cortisol Secretion and Adrenal Insufficiency

**DOI:** 10.1210/jendso/bvaf136

**Published:** 2025-08-20

**Authors:** Jasmine Saini, Bahaa Salama, Kai Yu, Shireen R Chacko, Ashley J Han, Camila Villavicencio Torres, Mohammad Hassan Murad, Irina Bancos

**Affiliations:** Division of Endocrinology, Diabetes, Metabolism, and Nutrition, Mayo Clinic, Rochester, MN 55905, USA; Department of Internal Medicine, Yale New Haven Hospital, New Haven, CT 06510, USA; Division of Endocrinology, Diabetes, Metabolism, and Nutrition, Mayo Clinic, Rochester, MN 55905, USA; Division of Endocrinology, Diabetes, Metabolism, and Nutrition, Mayo Clinic, Rochester, MN 55905, USA; Division of Endocrinology and Metabolism, West China Hospital of Sichuan University, Chengdu, Sichuan 610041, China; Division of Endocrinology, Diabetes, Metabolism, and Nutrition, Mayo Clinic, Rochester, MN 55905, USA; Department of Internal Medicine, Mayo Clinic, Rochester, MN 55905, USA; Division of Endocrinology, Diabetes, Metabolism, and Nutrition, Mayo Clinic, Rochester, MN 55905, USA; Evidence-based Practice Center, Mayo Clinic, Rochester, MN 55905, USA; Division of Endocrinology, Diabetes, Metabolism, and Nutrition, Mayo Clinic, Rochester, MN 55905, USA; Department of Laboratory Medicine and Pathology, Mayo Clinic, Rochester, MN 55905, USA

**Keywords:** adrenal, diagnosis, cortisol, sensitivity, specificity, likelihood ratio

## Abstract

**Context:**

Data on diagnostic accuracy of dehydroepiandrosterone sulfate (DHEA-S) for mild autonomous cortisol secretion (MACS) and adrenal insufficiency (AI) are discrepant.

**Objective:**

We conducted a systematic review and meta-analysis of published studies assessing the accuracy of DHEA-S in diagnosing MACS or AI.

**Methods:**

From inception to January 8, 2024, we searched databases for original studies of at least 20 participants with MACS or AI. MACS was defined as postdexamethasone cortisol greater than 1.8 mcg/dL or postsurgical hypocortisolism. AI was defined by abnormal dynamic testing. QUADAS-2 was used to assess the risk of bias. Bivariate random effects meta-analysis was used to generate pooled diagnostic accuracy estimates.

**Results:**

Seven studies on DHEA-S accuracy in diagnosing MACS (574 patients with MACS, 830 referent individuals), and 2 studies on DHEA-S accuracy in diagnosing AI (52 patients with AI, 59 referent individuals) were included. A meta-analysis of studies using DHEA-S cutoff between 60 and 70 mcg/dL to diagnose MACS demonstrated a sensitivity of 82% (95% CI, 64%-93%) and a specificity of 82% (95% CI, 74%-88%). In the 2 studies evaluating DHEA-S in diagnosing AI, the reference standard was a 1-mcg cosyntropin stimulation test. The sensitivity of DHEA-S for diagnosing AI ranged from 70.3% to 86.7%, and the specificity was 87.1%. Most studies were at a moderate risk of bias.

**Conclusion:**

Based on limited heterogeneous evidence, measurement of DHEA-S provides additional value in diagnosing MACS, as well as AI.

Dehydroepiandrosterone sulfate (DHEA-S) is secreted from the adrenal cortex in response to stimulation by adrenocorticotropin (ACTH) from the pituitary gland [1]. DHEA-S concentrations are very low or undetectable in patients with primary adrenal insufficiency (AI) due to the destruction of the adrenal gland cortex [2]. Reduced DHEA-S concentrations are also observed when the pituitary gland secretes less ACTH, as seen in central AI [3-6] or as a result of the negative feedback response in ACTH-independent hypercortisolism [7]. The diagnosis of AI and cortisol excess requires a high degree of clinical suspicion and is frequently supported by a combination of multiple biochemical tests to establish the diagnosis [8-11]. Morning cortisol concentration fluctuates widely and may be found to be normal in patients with adrenal disorders [12-14]. Therefore, dynamic tests such as the cosyntropin stimulation test (CST) and the dexamethasone suppression test (DST) are often required to diagnose AI and hypercortisolism, respectively [8, 15]. The concomitant measurement of DHEA-S in addition to cortisol may offer a faster and affordable approach in the evaluation of adrenal disorders.

Several studies have evaluated the accuracy of DHEA-S in AI; however, these were limited by small sample sizes as well as heterogeneity of assays, DHEA-S cutoffs, and reference standards [3-6]. Recent guidelines suggest that low DHEA-S concentrations are supportive of a diagnosis of mild autonomous cortisol secretion (MACS) [8]. However, most published studies on the diagnostic accuracy of DHEA-S have either not used the modern definition of MACS or have used variable assays and DHEA-S cutoffs, making strong conclusions and clinical application challenging.

Our objective was to conduct a systematic review and meta-analysis of the diagnostic accuracy studies of DHEA-S in MACS and AI.

## Materials and Methods

We conducted a systematic review following the recommendations of the Cochrane Collaboration for Systematic Reviews of Diagnostic Test Accuracy. The protocol was predefined, including a clear PICO (patient, intervention, comparison, outcome) question, and the reporting of this study follows the standard of Preferred Reporting Items for Systematic Reviews and Meta-analyses (PRISMA) [16]. Possible explanations of heterogeneity, such as various DHEA-S cutoffs and different assays for DHEA-S and cortisol, were considered prior to conducting the systematic review.

### Data Sources and Search Strategies

We conducted a comprehensive search of several databases from each database's inception to January 8, 2024, in all languages. The databases included Ovid MEDLINE and Epub ahead of print, in-process, and other nonindexed citations, and Daily, Ovid EMBASE, Ovid Cochrane Central Register of Controlled Trials, Ovid Cochrane Database of Systematic Reviews, and Scopus. Both search strategies were designed and conducted by a medical reference librarian (L.P.) with input from the principal investigator (I.B.).

Controlled vocabulary supplemented with key words was used to search for studies of the diagnostic accuracy of DHEA-S for MACS or AI in adults. The actual strategy listing all search terms used and their combinations is available in Supplementary Tables S1 and S2 [17]. Two independent reviewers conducted the screening in two phases: reviewing titles and abstracts (phase 1), followed by the selection of full-text manuscripts (phase 2). Disagreements were resolved by a third reviewer or via consensus.

### Study Selection

A comprehensive search of original studies, including randomized controlled trials, observational studies, and case series, assessing the diagnostic performance of DHEA-S were included. Only studies with a minimum sample size of 20 patients were included.

#### Dehydroepiandrosterone sulfate in mild autonomous cortisol secretion

MACS was defined as postdexamethasone cortisol greater than 1.8 mcg/dL or postadrenalectomy AI (reference standard). Postadrenalectomy AI is defined as serum cortisol less than 5 mcg/dL within the first few days after surgery or the need for glucocorticoid replacement. Postadrenalectomy AI is retrospective evidence for autonomous cortisol production. The comparison group included patients with adrenal adenoma without MACS or referent individuals without an adrenal mass. All cutoffs and assays of DHEA-S were included.

#### Dehydroepiandrosterone sulfate in adrenal insufficiency

AI was defined by positive testing from any of the following dynamic tests: CST, overnight metyrapone test, or insulin stimulation test. The comparison group included referent individuals.

### Data Extraction

#### **Dehydroepiandrosterone sulfate** in **mild autonomous cortisol secretion**

Data extraction (phase 3) was carried out in duplicate independently by 2 reviewers (S.C. and B.S.). The variables collected for each study included the name of the first author, year of publication, country where the study was conducted, study design, patient enrollment period, exclusion criteria, number of patients with MACS and referent group, number of women in MACS and referent group and their mean (SD) for age, assay, and cutoff for DHEA-S, reference standard, assay for cortisol, and diagnostic accuracy parameters (true positive, true negative, false positive, false negative, sensitivity, and specificity). Any discrepancies were resolved via consensus or a third reviewer (J.S. or I.B.).

#### Dehydroepiandrosterone sulfate in adrenal insufficiency

Data extraction was carried out by 2 independent reviewers (A.H. and C.V.). The variables collected were the name of the author, year of publication, study design, patient enrollment period, description of the cohort, exclusion criteria, number of patients with AI and referent group, number of women in AI and referent group and their mean (SD) for age, assay, and cutoff for DHEA-S, reference standard, assay of cortisol or 11-deoxycortisol, and diagnostic accuracy parameters (true positive, true negative, false positive, false negative, sensitivity, and specificity). Any discrepancies were resolved via consensus or a third reviewer (J.S. or I.B.).

### Risk of Bias Assessment and Certainty in Estimates

A duplicate and independent assessment for risk of bias and applicability of findings related to patient selection, reference, index test, and patient flow and timing was conducted using a QUADAS-2 tool tailored to our systematic review.

Patient selection was considered at a high risk of bias if the enrollment was not consecutive or random, if a case-control design was used, or if the study used inappropriate exclusions such as laterality, tumor size, type of nodularity, concomitant hormone secretion, or demographics. Index test (DHEA-S) was considered at high risk of bias if the threshold was not prespecified, if different DHEA-S assays were used, or if DHEA-S was interpreted with the knowledge of the overnight DST. The reference standard was at a higher risk of bias if the studies included any additional parameters to diagnose MACS/AI or if the comparison group included individuals without an adrenal adenoma. Flow and timing were given a higher risk of bias rating if all patients were not included in the analysis. The certainty in evidence (certainty in estimates) was assessed using the GRADE approach [18, 19].

### Statistical Analysis

Meta-analysis was conducted using a Bayesian bivariate hierarchical model using integrated nested Laplace approximations [20]. The sensitivity, specificity, positive and negative likelihood ratios, and diagnostic ratios were estimated with a 95% CI. Heterogeneity between studies was assessed using chi-square and *I^2^*. All analysis was conducted using R software macOS (version 4). Figure for risk of bias assessment and certainty in estimates was created using the robvis (visualization tool) [21].

## Results

### Accuracy of Dehydroepiandrosterone Sulfate in Diagnosing Mild Autonomous Cortisol Secretion

#### Inclusion and description of studies

**Figure 1.**
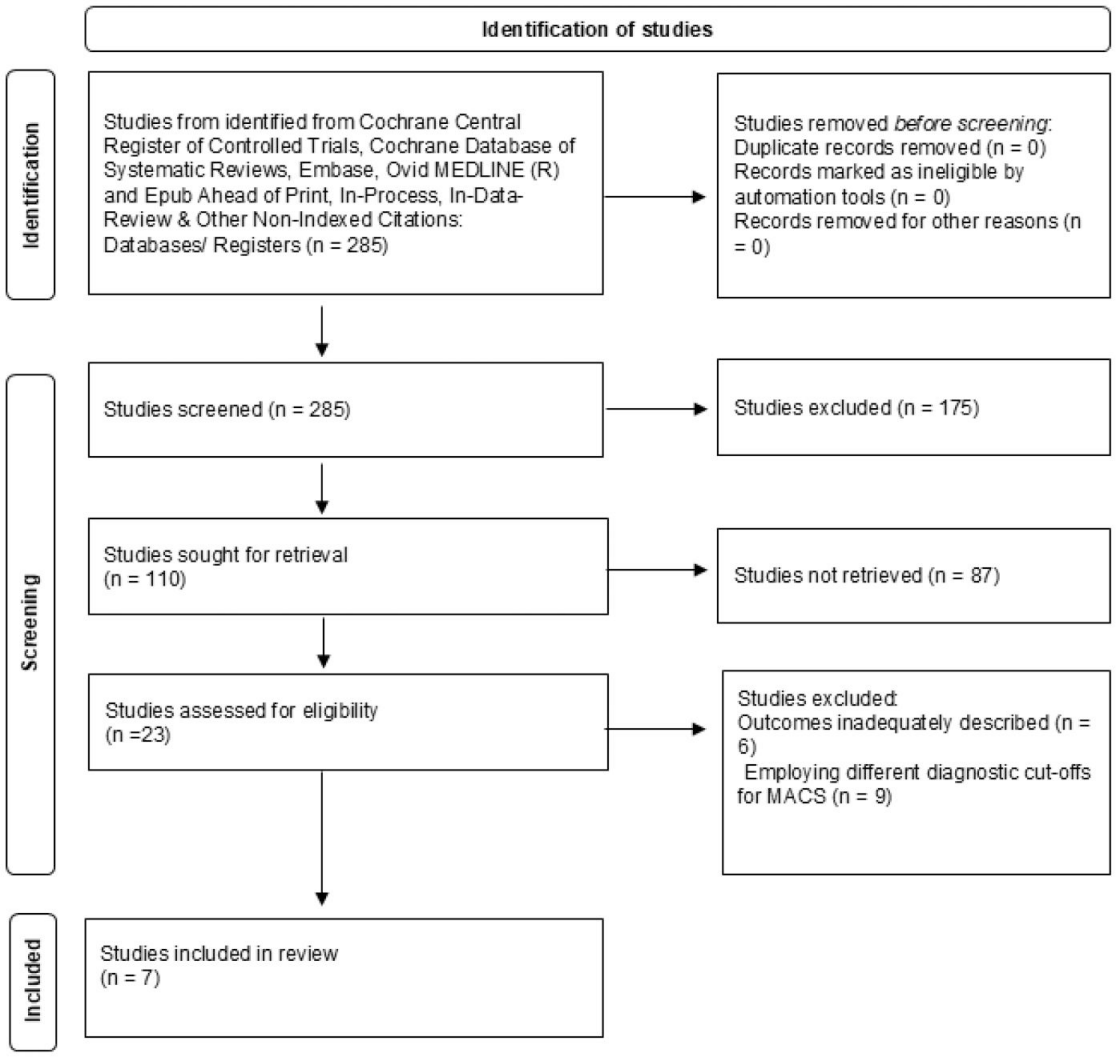
PRISMA flowchart for assessment of diagnostic accuracy of dehydroepiandrosterone sulfate in mild autonomous cortisol secretion.

The systematic search through several databases identified a total of 285 references. Of 285 references, 175 studies were excluded after abstract and title screening. The remaining 110 studies were sought for retrieval, and 87 studies were excluded after the full-text review. The remaining 23 studies [7, 22-43] were assessed for eligibility, and ultimately, 7 studies [7, 22, 23, 31, 33, 35, 42] were included in the full-text extraction after excluding studies with inadequately described outcomes or those employing different diagnostic cutoffs for MACS, Fig. 1.

The demographic characteristics of the included studies are described in Table 1. The 7 studies (8 cohorts) consisted of a total of 574 patients with MACS and 830 individuals in the referent group (including nonfunctioning adenoma). The mean age was specified in only 5 studies (6 cohorts) with similar distribution among patients (55.5 years) and referent groups (53.3 years) (see Table 1). However, the patients with MACS had a higher proportion of women (323/472, 68.4%) compared to the referent group (352/658, 53.4%).

**Table 1.** Demographic characteristics of studies for diagnostic accuracy of dehydroepiandrosterone sulfate in mild autonomous cortisol secretion

Author, y	Country of publication	Study design	Enrollment period	Patients with MACS			Referent individuals			
				n	Mean age (SD)	n with DHEA-S available	n	Mean age (SD), y	n with DHEA-S available	Type of referent individuals
Carafone, 2021 (7)	USA	Retrospective cohort	1/1/2020-4/20/2020	256	54.7 (13)	256	212	53.3 (13.3)	212	NFA
Ueland, 2020 (42)	Norway	Retrospective cohort	6/2021-8/2018	83	60 (12.6)	58	82	61.2 (10.9)	54	NFA
Akehi, 2013 (22)	Japan	Retrospective cohort	4/2003-8/2011	26	—	26	51	—	51	NFA
Araujo-Castro, 2021 (23)	Spain	Retrospective cohort	2013-2020	76	—	76	121	—	121	NFA
Hana, 2019 (31)	Czech Republic	Cross-sectional	9/2013-6/2017	41	65.5 (5.3)	41	11	55.3 (12.9)	11	NFA
Lee, 2017 (33)	South Korea	Prospective cohort	7/2011-6/2014	33	49.8 (10)	33	66	47.4 (10.9)	66	NFA
Liu, 2022 (development cohort) (35)	China	Prospective cohort	10/2019-4/2021	45	53 (13)	45	242	53.2 (12.9)	242	NFA
Liu, 2022 (validation cohort) (35)	China	Prospective cohort	5/2021-9/2021	14	50.3 (8.5)	14	45	49.4 (10.1)	45	NFA

Abbreviations: DHEA-S, dehydroepiandrosterone sulfate; MACS, mild autonomous cortisol secretion; NFA, nonfunctioning adenoma.

In 5 of 7 studies (or 6/8 cohorts), the DHEA-S cutoff was predefined, and these cutoffs were heterogeneous across the studies [22, 23, 31, 33, 35, 42]. Of 7 studies, 5 studies (or 6 cohorts) specified the DHEA-S assay, and 6 studies (or 7 cohorts) specified the cortisol assay (Table 2). All studies except one [33], used 1-mg DST as the reference standard with a cutoff of 1.8 mcg/dL [8] (see Table 2).

**Table 2.** Index and reference test characteristics of studies included in the final data extraction for diagnostic accuracy of dehydroepiandrosterone sulfate in mild autonomous cortisol secretion

Author	DHEA-S cutoff, mcg/dL	Was DHEA-S cutoff predefined?	DHEA-S assay	Reference standard	Cortisol assay
Carafone, 2021 (7)	<15	No	Chemiluminescent immunoenzymatic assay (Access Cortisol, Beckman-Coulter, 2007)	1 mg DST > 1.8 mcg/dL	Competitive binding immunoenzymatic assay (Access Cortisol, Beckman-Coulter, 2007)
	<25				
	<40				
	<50				
	<80				
	<100				
Ueland, 2020 (42)	<40	Yes	Chemiluminescent immunoassay (Siemens Immulite 2000 XPi)	1 mg DST > 1.8 mcg/dL	Liquid chromatography–mass spectrometry
Akehi, 2013 (22)	Age- and sex-based range	Yes	Not reported	1 mg DST > 1.8 mcg/dL	Electrochemiluminescence immunoassay (Elecsys 2010; Roche)
Araujo-Castro, 2021 (23)	Age- and sex-based range	Yes	Immunochemiluminescence assay	1 mg DST > 1.8 mcg/dL	Immunochemiluminescence assay (Architect i2000 systems Abbott Diagnostics platform)
Hana, 2019 (31)	<67.5	Yes	Gas chromatography–mass spectrometry	1 mg DST > 1.8 mcg/dL	Radioimmunoassay
Lee, 2017 (33)	<80: men; <35: women	Yes	Not reported	Postsurgical hypocortisolism	Not reported
Liu, 2022 (development cohort) (35)	<40	No	Direct chemiluminescent immunoassay (Siemens Atellica)	1 mg DST > 1.8 mcg/dL	Direct chemiluminescent immunoassay
	<50				
	<60				
	<90				
	<120				
Liu, 2022 (validation cohort) (35)	<60	Yes		1 mg DST > 1.8 mcg/dL	

Abbreviations: DHEA-S, dehydroepiandrosterone sulfate; DST, dexamethasone suppression test.

All DHEA-S assays used chemiluminescent immunoassays except that by Hana et al [31], which employed gas chromatography–mass spectrometry. Similarly, for the cortisol assays, most studies employed chemiluminescent immunoassay with two exceptions: Ueland et al using liquid chromatography–mass spectrometry and Hana et al using radioimmunoassay [31, 42].

#### Accuracy of dehydroepiandrosterone sulfate in diagnosing mild autonomous cortisol secretion

**Figure 2.**
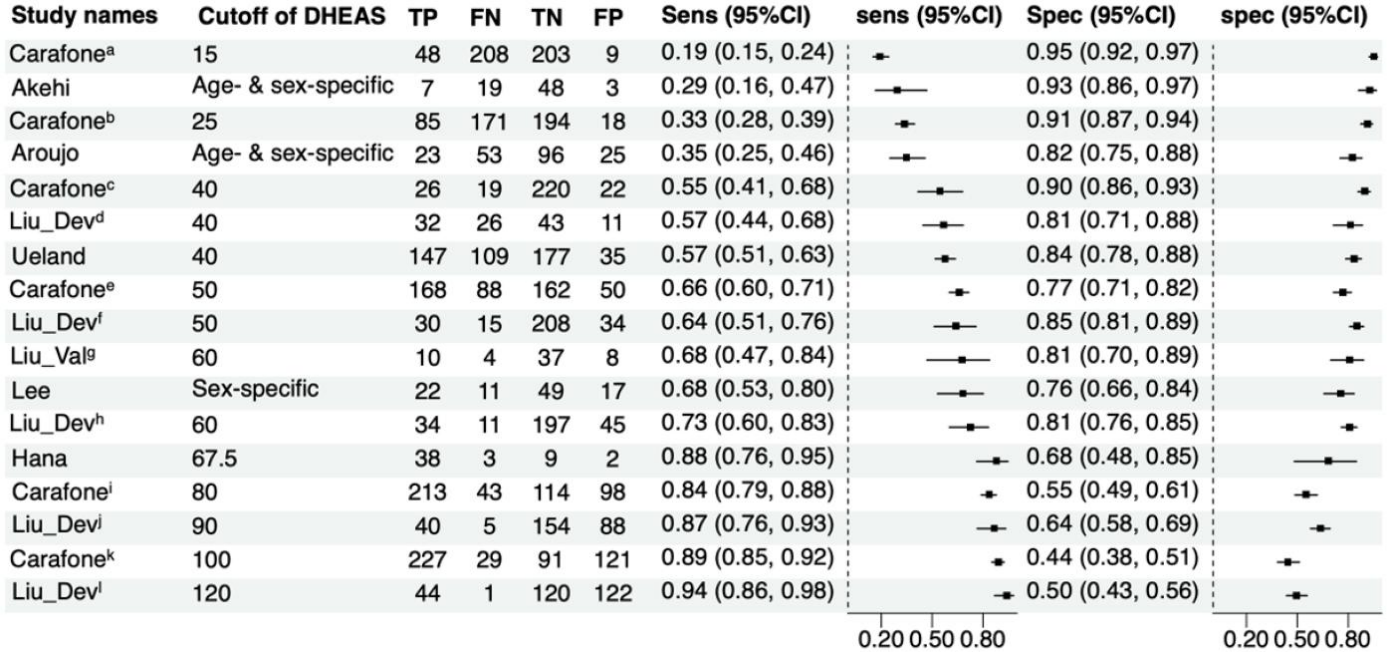
Forest plot of sensitivity and specificity of individual studies for assessment of accuracy of dehydroepiandrosterone sulfate (DHEA-S) in mild autonomous cortisol secretion (MACS).

The sensitivity of DHEA-S for diagnosing MACS was low at DHEA-S cutoffs between 15 and 25 mcg/dL, ranging from 19% to 33%, and high at DHEA-S cutoffs greater than 60 mcg/dL, ranging from 73% to 94% (Table 3 and Fig. 2). Conversely, specificity was highest at DHEA-S cutoffs of 40 mcg/dL or less, ranging from 81% and 95% and only 44% to 50% at DHEA-S of 100 mcg/dL or greater (see Fig. 2).

**Table 3.** Diagnostic accuracy parameters for included studies for diagnostic accuracy of dehydroepiandrosterone sulfate in mild autonomous cortisol secretion

Author, y	DHEA-S cutoff, mcg/dL	Year	TP	TN	FP	FN	Sensitivity, %	Specificity, %
Carafone, 2021 (7)	15	2021	48	203	9	208	18.8	95.8
	25		85	194	18	171	33.2	91.5
	40		147	177	35	109	57.4	83.5
	50		168	162	50	88	65.6	76.4
	80		213	114	98	43	83.2	53.8
	100		227	91	121	29	88.7	42.9
Ueland, 2020 (42)	40	2020	32	43	11	26	55.2	79.6
Akehi, 2013 (22)	Age- and sex-based	2013	7	28	3	19	26.9	94.1
Araujo-Castro, 2021 (23)	Age- and sex-based	2021	23	96	25	53	30.3	79.3
Hana, 2019 (31)	67.5	2019	38	9	2	3	92.7	81.8
Lee, 2017 (33)	Age- and sex-based	2017	22	49	17	11	66.7	74.2
Liu (development cohort) 2022 (35)	40	2022	26	220	22	19	57.8	90.9
	50		30	208	34	15	66.7	86
	60		34	197	45	11	75.6	81.4
	90		40	154	88	5	88.9	63.6
	120		44	120	122	1	97.8	49.6
Liu (validation cohort), 2022 (35)	60	2022	10	37	8	4	71.4	82.2

Abbreviations: DHEA-S, dehydroepiandrosterone sulfate; FN, false negative; FP, false positive; TN, true negative; TP, true positive.

**Figure 3.**
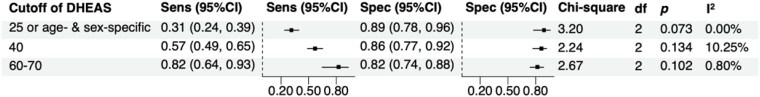
Meta-analysis of diagnostic efficacy of dehydroepiandrosterone sulfate (DHEA-S) in mild autonomous cortisol secretion (MACS) at different cutoff values: pooled sensitivity and specificity.

Meta-analysis of studies investigating DHEA-S cutoffs of 60 to 70 mcg/dL demonstrated the best accuracy estimated for diagnosing MACS (pooled sensitivity of 82% [95% CI, 64%-93%] and pooled specificity of 82% [95% CI, 74%-88%]) (Fig. 3).

#### Methodological quality and certainty in the estimates

**Figure 4.**
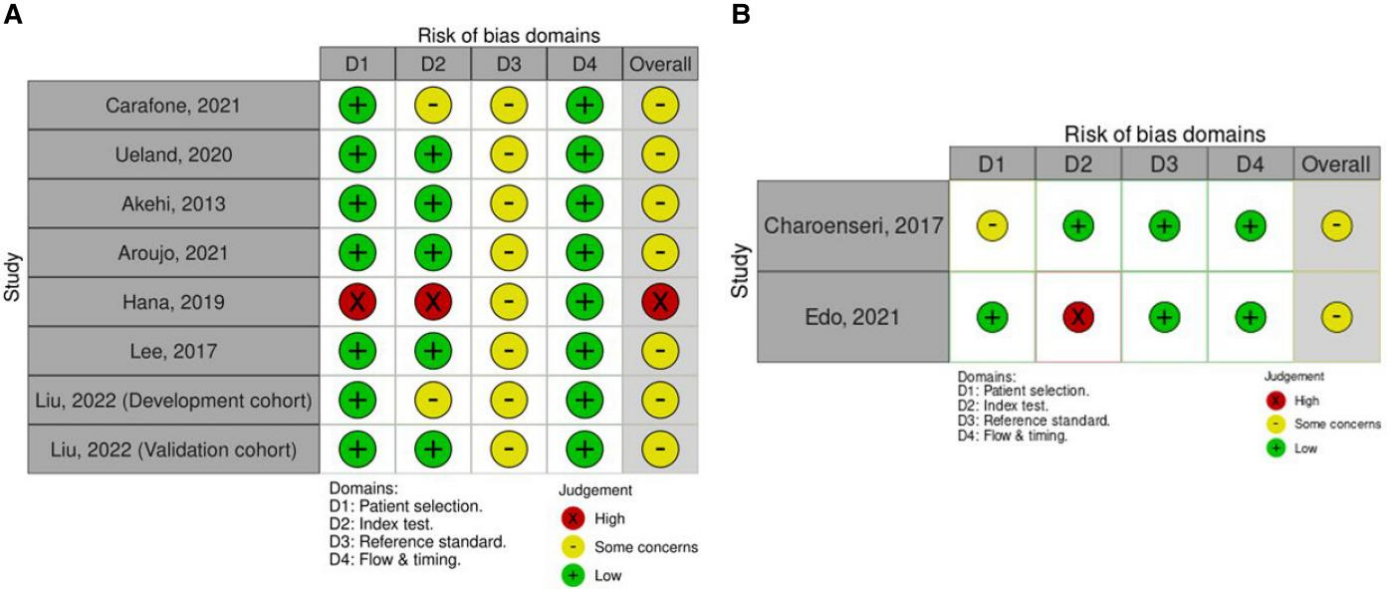
Risk of bias assessment using the QUADAS-2 tool for diagnostic accuracy of A, dehydroepiandrosterone sulfate (DHEA-S) in mild autonomous cortisol secretion (MACS) and B, adrenal insufficiency (AI).

The risk of bias assessment was conducted using the QUADAS-2 tool tailored to the study, (Fig. 4 and Supplementary Table S3 [17]). Limitations of the studies included a lack of consecutive patient enrollment and exclusion of patients with other hormonal excess or specific demographic characteristics, which increased the risk of bias in patient selection. Other limitations were the inclusion of patients with nonfunctioning adenoma as a referent group and not predefining the DHEA-S cutoff. Most studies had a high risk of bias for the index and reference standard, whereas the concern for flow and timing was low. The certainty in the meta-analytic estimates was low, considering the increased risk of bias and imprecision (small sample size).

### Dehydroepiandrosterone Sulfate Accuracy in Diagnosing Adrenal Insufficiency

#### Inclusion and description of studies

**Figure 5.**
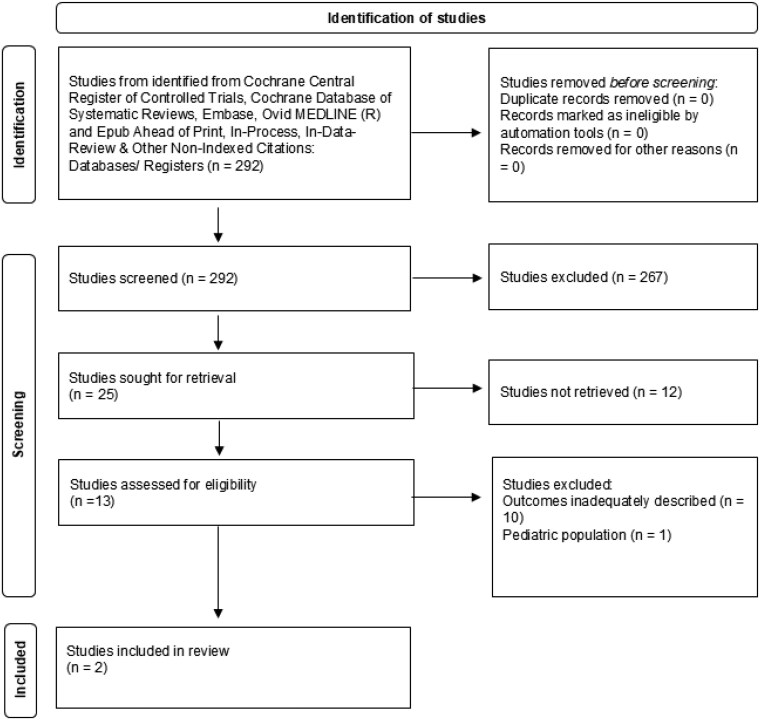
PRISMA flowchart for assessment of diagnostic accuracy of dehydroepiandrosterone sulfate (DHEA-S) in adrenal insufficiency (AI).

A systematic search through various databases yielded a total of 292 references. Of 292 references, 267 were excluded during the abstract and title screening (phase 1 of screening). The remaining 25 studies were sought for retrieval, and 13 studies were excluded after a full-text review (phase 2 of screening). Finally, 12 studies [3, 4, 6, 44-52] were assessed for eligibility and 2 studies [3, 4] were included in the final data extraction, after excluding 11 studies due to inadequately described outcomes or including the pediatric population (Fig. 5).

The demographic details and diagnostic accuracy of the included studies are illustrated in Table 4. We included a total of 2 studies, comprising a total of 52 patients with AI and 59 referent individuals. Only Edo et al [4] specified the demographics, such as age (56.7 years, SD 17.4) and sex proportion (women n = 19, 67.9%) (see Table 4). Charoensri et al used a predefined DHEA-S cutoff and Edo et al defined the DHEA-S cutoff post analysis [3, 4]. Low-dose CST with post-CST cortisol cutoff of less than 18 mcg/dL was used as the reference standard. Chemiluminescence immunoassay was used for the DHEA-S and cortisol assay in both studies.

**Table 4.** Demographic characteristics and diagnostic accuracy of studies included for diagnostic accuracy of dehydroepiandrosterone sulfate in adrenal insufficiency

Characteristics		Author, y	
		Charoensri, 2017	Edo, 2021
Country		Thailand	Japan
Study design		Cross-sectional study	Retrospective cohort study
Enrollment period		Not specified	04/2014-01/2020
Total sample size		46	65
Patients with AI	N	15	37
	Women, n (%)	Not specified	18 (48.6%)
	Mean age (SD)	Not specified	56.7 (17.4)
	N with DHEA-S available	15	37
Referent individuals	N	31	28
	Women, n (%)	Not specified	19 (67.9%)
	Mean age (SD)	Not specified	52.6 (16.7)
	N with DHEA-S available	31	28
DHEA-S cutoff		Normal age- and sex- specific	SD of log-transformed DHEA-S = 0.853
Was DHEA-S predefined?		Yes	No
DHEA-S assay		Solid-phase competitive chemiluminescent enzyme assay	Chemiluminescence enzyme immunoassay
Reference standard		1 mcg post-CST cortisol < 18 mcg/dL	1 mcg post-CST cortisol < 18 mcg/dL
Cortisol assay		Immunoassay using chemiluminescent technique	Electrochemilumiscence immunoassay
Diagnostic accuracy parameters	True positive	13	26
	True negative	27	28
	False positive	4	0
	False negative	2	11
	Sensitivity, %	86.7	70.3
	Specificity, %	87.1	87.1

Abbreviations: AI, adrenal insufficiency; CST, cosyntropin stimulation test; DHEA-S, dehydroepiandrosterone sulfate.

#### Diagnostic accuracy

Based on the cutoffs in the 2 studies, the sensitivity of DHEA-S for diagnosing AI ranged from 70.3% to 86.7% and specificity was 87.1% in both studies. However, we were unable to conduct a meta-analysis due to a limited number of studies. Heterogeneity was not explored due to the insufficient number of studies.

#### Methodological quality and certainty in the estimates

Most studies had a moderate to high risk of bias for index and reference tests, whereas the concern for flow and timing was low (see Fig. 4 and Supplementary Table S4 [17]**)**. The certainty in the meta-analytic estimates was low, considering the increased risk of bias and imprecision (small sample size).

## Discussion

We conducted a systematic review of studies reporting on the accuracy of DHEA-S in diagnosing MACS or AI. Meta-analysis of studies investigating DHEA-S in MACS demonstrated that a DHEA-S cutoff of less than 60 to 70 mcg/dL performed best, with a pooled sensitivity and specificity of 82%. Lower DHEA-S cutoffs had a slightly better sensitivity of 86% to 89% but a much lower sensitivity of 31% to 57% for the diagnosis of MACS. We found only 2 small studies investigating the accuracy of DHEA-S in diagnosing AI, reporting a sensitivity of 70% to 87% and specificity of 87%.

As expected, we found that a lower DHEA-S cutoff was associated with lower sensitivity and higher specificity in diagnosing MACS. We were unable to explore the effect of various DHEA-S and cortisol assays on the diagnostic accuracy of DHEA-S due to the limited number of studies and patients in each group. As the reference standard was based on postdexamethasone cortisol in patients with MACS, misclassification was possible due to potential false-positive results reported in 3% to 20% of patients [53]. In the 2 small studies assessing the diagnostic accuracy of DHEA-S in AI, the reference standard was the low-dose (1-mcg) CST. Low-dose CST was reported to have a sensitivity of 83% and specificity of 86% in diagnosing secondary AI when compared to the gold standard of insulin tolerance test and overnight metyrapone test, thus potentially underdiagnosing AI [10]. A recent study that was published after the timeline listed in our protocol investigated the accuracy of DHEAS in diagnosing AI, with a reference standard of 250-mcg CST. In this study, DHEAS demonstrated a good diagnostic performance with an area under the curve of 0.81 (95% CI, 0.78-0.85) [54].

The overall methodological quality of studies included in this systematic review, as evaluated by the QUADAS-2 tool, was moderate. The risk of bias was low for most studies in the patient selection, flow, and timing domains. However, the risk of bias was higher in the index test and reference test assessments. While all studies used the postdexamethasone cortisol greater than 1.8 mcg/dL, none of the studies had referent individuals as people without adrenal disorders. Risk of bias was variable in the index test assessment, mainly due to a lack of predefining the DHEA-S cutoff. Additionally, the estimates were drawn from a relatively small sample size with wide CIs.

Strengths of this study include a comprehensive literature search by an experienced librarian, a search using multiple databases, duplicate assessment at screening, data extraction, methodological quality assessment, predefined protocol questions, and a priori hypotheses for exploring heterogeneity. Both systematic reviews conducted have various limitations, such as variability of DHEA-S and cortisol assays, heterogeneous reference standards, potential false-positive postdexamethasone cortisol results misclassifying patients with MACS, a lack of patients without adrenal diseases as referent individuals, and a limited number of studies or limited pooled subgroup analysis, which lowers the confidence in the estimated effects.

In conclusion, based on limited data, the DHEA-S cutoff of 60 to 70 mcg/dL has a sensitivity and specificity of 82% in diagnosing MACS. Based on only 2 studies in our systematic review, and a larger, more recent study of patients with AI, DHEA-S adds value in AI diagnosis. Further studies with an optimal reference standard, larger sample size, and robust methodology are needed to generate confident estimates of accuracy of DHEAS in diagnosing both MACS and AI.

## Data Availability

Some or all data sets generated during or analyzed during the current study are not publicly available but are available from the corresponding author on reasonable request.

## Abbreviations

ACTH: adrenocorticotropin
AI: adrenal insufficiency
CST: cosyntropin stimulation test
DHEA-S: dehydroepiandrosterone sulfate
DST: dexamethasone suppression test
MACS: mild autonomous cortisol secretion
